# Diagnosing coeliac disease in the elderly: a United Kingdom cohort study 

**Published:** 2020

**Authors:** Mohamed G Shiha, Lauren J Marks, David S. Sanders

**Affiliations:** *Academic Unit of Gastroenterology, Royal Hallamshire Hospital, Sheffield, United Kingdom*

**Keywords:** Coeliac Disease, Elderly, Gluten.

## Abstract

**Aim:**

To assess the outcomes for an elderly population with coeliac disease and to compare with younger adults with CD.

**Background:**

Coeliac disease in the elderly has been underdiagnosed due to the heterogeneity of presentation as well as lack of physicians’ awareness of CD in this population. However, the benefits of diagnosing CD in the elderly may be contentious.

**Methods:**

Newly diagnosed CD patients were prospectively recruited from the Coeliac Specialist Clinic at the Royal Hallamshire Hospital, Sheffield, between 2008 and 2017. All patients had villous atrophy (VA) on biopsy with positive coeliac serology. Additionally, the patients were retrospectively recruited from 1990 to 2008 to determine the trend in elderly CD diagnostic frequency over time.

**Results:**

A total of 1605 patients with CD were recruited (n=644 prospectively, n=961 retrospectively). Of these, 208 patients (13.0%) were diagnosed over the age of 65 years between 1990 and 2017. The proportion of elderly CD diagnoses increased from 0% in 1990-1991 to 18.7% in 2016-2017 (p<0.001). Younger patients more commonly presented with fatigue (p<0.001) and gastrointestinal symptoms including diarrhoea (p=0.005), abdominal pain (p=0.019), and IBS-type symptoms (p=0.008), while older people more frequently presented with B12 deficiency (p=0.037).

**Conclusion:**

The prevalence of CD in the elderly has significantly increased over the last two decades, but elderly patients tend to present with fewer symptoms. Further research is required to determine whether a strict gluten-free diet in these patients is a necessity or a burden.

## Introduction

 Coeliac Disease is an autoimmune enteropathy in which genetically susceptible individuals experience chronic small intestinal inflammation on ingestion of dietary gluten (1) .

Until the 1980s, CD was considered to be a rare enteropathy exclusively affecting paediatric patients, with malabsorptive features manifesting around the time of weaning. “Classical” clinical signs included chronic diarrhoea, weight loss, and failure to thrive (2) . However, the last four decades have observed a striking shift in the epidemiology and clinical presentation of CD. Current studies demonstrate a four-fold increase in the disease prevalence over the last 22 years (2) , with a total prevalence of 0.7 – 2% (3) (4) . 

CD in the elderly has been underdiagnosed due to the lack of physicians’ awareness of CD occurrence in this age group and the heterogeneity of presentation. Evidence suggests that a remarkable number of patients have been misdiagnosed with IBS several years prior to CD diagnosis. This has caused an average delay of 17 years before the correct diagnosis was made (5) .

Elderly patients presenting with CD symptoms that can also denote malignancy, such as anaemia and weight loss often result in a diagnostic work-up for gastrointestinal neoplasia prior to considering CD. When mild and not suggestive of malignancy, symptoms such as alterations in bowel habits can be accredited to a functional aetiology, such as irritable bowel syndrome (IBS), psychiatric conditions including anxiety and depression or a by-product of the typical ageing process (6) .

While elderly CD patients have no increase in mortality when compared to the general population (7)  (8) , they may suffer from subclinical malabsorption (9) , reduced bone density, and increased risk of fractures (10) . Furthermore, CD patients have a 6- to 9-fold higher risk of enteropathy-associated T-cell lymphoma and non-Hodgkin lymphoma than the general population (11) (12) . A recent meta-analysis found that CD patients are at a statistically significant increased risk of oesophageal and small bowel carcinoma but the prevalence of other GI cancers, such as liver, pancreatic, gastric, and colorectal were comparable to the general population (13) . 

Numerous studies have demonstrated the protective effect of a GFD against malignancy (14) (15) (16) , with poor adherence being associated with increased risk of malignancy particularly of the mouth, pharynx and oesophagus as well as lymphoproliferative malignancy (14) .

It has been reported that the restrictions of a lifelong GFD amplify disease burden and reduce the quality of life (17) . This begs the question as to whether or not it is appropriate to pursue a CD diagnosis in the elderly, particularly if symptoms are subtle (18) . Elderly patients can be especially prone to low adherence due to long-established dietary habits that may prove difficult to change (18) , mainly in screen-detected subjects who are asymptomatic and thus do not experience a clinical benefit. Nonetheless, studies have shown that the majority of elderly CD patients have good adherence to strict GFD along with symptomatic improvement (19) (20)  and mucosal remission (7) . Surprisingly, Vilppula et al. reported that GFD did not worsen quality of life in elderly CD patients (7) . A possible explanation for this is that a number of CD patients who initially report no symptoms actually feel better after starting GFD (21) .

In this cohort study, we retrospectively examined the trend in elderly CD diagnostics in Sheffield from 1990 to 2008. Additionally, to accurately assess the variant clinical presentations, we prospectively recruited newly diagnosed CD patients between 2008 and 2017. Our aim was to determine the prevalence of newly diagnosed coeliac disease in patients over the age of 65 and to assess the outcomes for an elderly population in comparison with younger adults with CD. 

## Methods

A tertiary centre cohort study of adult coeliac disease patients diagnosed at Sheffield Teaching Hospitals NHS foundation trust was conducted using a combination of prospective and retrospective data. Between 1990 and 2017, 1605 patients received a diagnosis of coeliac disease, and this population was used to measure the prevalence of elderly CD over these three decades.

In order to accurately investigate variations in clinical presentation of coeliac disease with diagnostic age, a smaller study cohort was used. Only prospectively recruited patients (those diagnosed from 2008 onwards) were considered for analysis (n=644).

Diagnoses made prior to 2008 were retrieved retrospectively through paper archives of outpatient clinic letters, with a subsequent search of the Royal Hallamshire Hospital Gastroenterology Shared Drive using the key term “coeliac disease” to ensure that no diagnoses were missed. The diagnosis of coeliac disease was checked against the diagnostic criteria for that time period.

Since the establishment of the Coeliac Specialist Clinic at the Royal Hallamshire Hospital in 2008, patients have been prospectively recruited with routine performance of duodenal biopsies, HLA genotyping, and serological tests including haematological, immunological, and biochemical markers. These data were combined with details of demographics, clinical presentation, and past medical history to create the final dataset. Data were introduced into a specialist Coeliac database, which received ethical approval from the Yorkshire and the Humber – Sheffield Research Ethics Committee (REC reference: 14/YH/1216).

Patients were required to be over the age of 16 years and have a definitive, histologically-confirmed diagnosis of coeliac disease. Patients were excluded from the study if they had been diagnosed with CD before the age of 16 years, tested negative for IgA-tTG and IgA-EMA antibodies, or did not have villous atrophy on biopsy.

All upper GI endoscopies were conducted using PENTAX gastroscopes (PENTAX Medical, Tokyo) between 2008 and 2017. Between 1 and 4 single bite biopsies were taken from the distal duodenum (D2) depending on the endoscopist performing the procedure. Where all 4 D2 biopsies were obtained, these were taken at the 3 o’clock, 6 o’clock, 9 o’clock and 12 o’clock positions. In the majority of cases (73%), an additional biopsy was collected from the duodenal bulb (D1). 

The date of diagnosis was defined as the date of the first gastroscopy and biopsy retrieval procedure in which villous atrophy was identified. 

EMA status and TTG values within 3 months of the date of diagnosis were taken as the presenting results. In cases where coeliac blood tests had been repeated within this period, patients were considered to be EMA positive at presentation if they had any degree of EMA positivity (weak positive, positive, or strong positive) on one or more serological tests in this period (22) . The highest TTG value obtained during this period was accepted as the presenting antibody level.

Similarly, haematological and biochemical parameters measured three months either side of the diagnosis date were considered to be the presenting values. Wherever multiple tests were carried out within this time period, the lowest value for each parameter was accepted as the presenting value. 

In order to compare the clinical phenotype of CD between the elderly and the young, patients were divided into three age groups. Patients were considered to be elderly if they were over 65 years of age. These age groups are as follows:

**Table 1:** Age group categorisation

Age group	Number of patients in age group	% of cohort in age group
Group 1: 16-34 years	258	40.1
Group 2: 35-64 years	287	44.6
Group 3: >65 years	99	15.4

**Statistical analysis**

Categorical variables were analysed using the Pearson Chi-Square Test of Association. Binary Logistic Regression was performed for categorical variables when accounting for covariates.

Continuous variables were assessed using a one-way univariate analysis of covariance (ANOVA). A univariate analysis of covariance (ANCOVA) was undertaken when accounting for covariates. Any continuous variables violating ANOVA assumptions were assessed using a Kruskal-Wallis non-parametric H test.

P-values were conducted at a base significance level of 0.05 and were two-tailed. This significance level was adjusted using the Bonferroni correction if multiple testing was undertaken. Where the dependent variable was ordinal, rather than binary, ordinal regression was performed. However, this test does not allow the consideration of co-variates.

Post-hoc pairwise testing was undertaken if any significant association was found between the age groups. A Bonferroni correction was applied to any pairwise tests. Post-hoc testing was performed using the same statistical method used for the broad analysis of the variable. It has one exception if the Kruskal-Wallis H test was used on the broad analysis. In this case, a Mann-Whitney U test was performed for post-hoc analysis. 

## Results

The number of diagnoses of coeliac disease in the elderly has increased dramatically over the years, particularly since 1998. However, elderly coeliac disease still accounts for only a minority of cases; CD diagnoses were significantly less common in the elderly than those under 65 (p<0.001). Spearman’s rank analysis of time against number of elderly diagnoses indicated a statistically significant strongly positive correlation (r_s_=0.883, p=<0.001).

**Figure 1:**
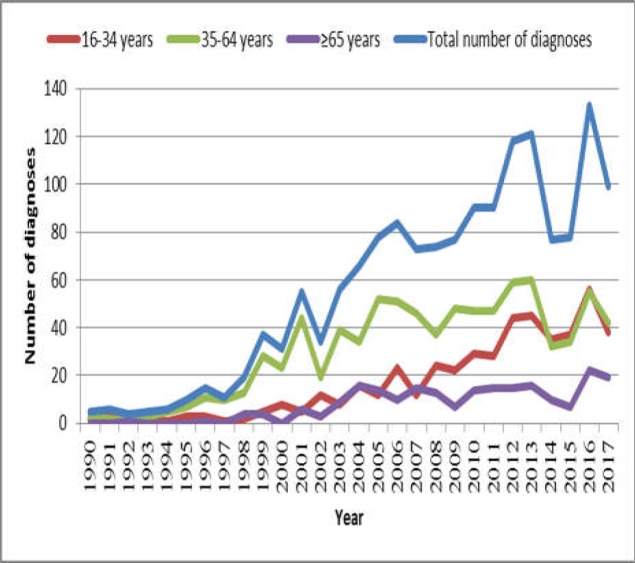
Line graph illustrating the number of coeliac disease diagnoses in Sheffield over time both in total and by age group

Figure 1 illustrates the ascending trend in CD diagnoses over time in the general population of Sheffield from 1994 to present, with particular acceleration in the rate of diagnosis after 1997. Diagnoses increased in all age groups over the last three decades. Age of 35-64 years is consistently the most common age of diagnosis up until 2014, from which point the proportion of patients aged 16-34 and 35-64 was comparable. The number of diagnoses in the over 65 and 16-34 age groups remained relatively similar with minor fluctuations until 2007, at which point there was a divergence in the number of diagnoses. The number of CD diagnoses made in the 16-34 age group escalated from 2007 onwards, whilst aged 65 and over remained relatively constant. Interestingly, there was a substantial drop in diagnoses from 2014 and 2015, with just 77 and 78 diagnoses made in each year respectively, compared to 121 in 2013.

**Clinical presentations**

We categorised the clinical presentation of CD as either malabsorption or non-malabsorption features regardless of “classical” or “non-classical” nomenclature (Table 2 and Table 3).The proportion of patients presenting with at least one malabsorption feature in the study population was 75.6% (n=487). The presence of malabsorption features varied from 61.5% in the 65 years and over age group to 67.5% in the 16-64 age groups (p= 0.339).

Diarrhoea and iron deficiency anaemia were the most common presenting features. The prevalence of diarrhoea was highest among the 16-34-year age group (p=0.005) while there was no significant association between the presence of iron deficiency anaemia at diagnosis and age of presentation (p=0.312)

Low ferritin without anaemia was present in 19.9% of the study population, ranging from 12.1% in the 16-34 age group to 22.5% in the elderly age group at diagnosis (p=0.222).

The prevalence of B12 deficiency was twice as common in the elderly group (**≥**65 years) compared to the younger age groups (p=0.037).

**Table 2:** Association between malabsorption features at presentation and age of CD diagnosis

Malabsorption feature	Prevalence in overall cohort (n=644)	Age (years)			p-value
		16-34 years (n=259)	35-64 years (n=287)	≥65 years (n=99)	
At least one malabsorption feature	67.5% (391)	67.5% (158)	69.8% (178)	55.6%(55)	0.339
Diarrhoea	30.4% (196)	35.7% (92)	30.0% (86)	18.2%(18)	0.005
Weight loss	7.9%(51)	8.5%(22)	7.3%(21)	8.1%(8)	0.807
Iron-deficiency anaemia (IDA)	34.9%(225)	34.5%(89)	37.3% (107)	29.3%(29)	0.312
B12 deficiency	12.1%(78)	10.5%(27)	10.8%(31)	20.2%(20)	0.037
Folate Deficiency	24.4%(157)	20.2%(52)	28.6%(82)	23.2%(23)	0.071
Low ferritin in isolation	19.9%(128)	22.5%(58)	20.2%(58)	12.1%(12)	0.222

**Table 3:** Association between non-malabsorption features at presentation and age of CD diagnosis

Non-malabsorption feature	Prevalence in overall cohort(n=644)	Age (years)			p-value
		16-34 years (n=259)	35-64 years (n=287)	>=65 years (n=99)	
Fatigue	24.9%(160)	31.9%(82)	23.0% (66)	12.1%(12)	<0.001
Abdominal pain	23.2%(149)	29.2%(75)	20.2%(58)	16.2%(16)	0.019
IBS- type symptoms	18.0%(116)	24.4%(63)	15.0%(43)	10.1%(10)	0.008
Osteopenia / osteoporosis	6.5%(42)	1.9%(5)	4.9%(14)	23.2%(23)	<0.001

Weight loss was a relatively uncommon presenting feature, being experienced by only 7.9% (n=51) of the study subjects and was very consistent across all age groups. 

Fatigue was present in 24.9% (n=160) of the study cohort. The prevalence of this extraintestinal feature varied from 12.1% in those diagnosed at age 65 or over to 31.9% in those aged 16-34 at diagnosis (p<0.001). 

Abdominal pain was significantly more prevalent in patients diagnosed at a younger age than those diagnosed at 65 years or over. The prevalence of abdominal pain amongst the study subjects was 23.2% (n=149), ranging from 16.2% in the ≥65 age group at diagnosis to 29.2% in the 16-34 diagnostic age group (p=0.019).

IBS-type symptoms were prevalent in 18.0% (n=116) of the study cohort, varying from 10.1% in those diagnosed at age 65 or over to 24.4% in those diagnosed aged 16-34 years. This difference was significant when comparing the elderly age group to both the 16-34 (p=0.001) and 35-64 (p=0.025) age groups.

Osteopenia and osteoporosis at prompted referral for CD investigation were observed in 6.5% (n=42). Unsurprisingly, the majority of patients presenting with reduced bone mineral density were diagnosed over the age of 64 years old (23.2%). Presentation with osteopenia or osteoporosis under the age of 65 years was rare, with only 1.9% and 4.9% experiencing this in the 16-34- and 35-64-year age groups respectively (p<0.001). This suggests a statistically significant association between osteopenic/osteoporotic presentation and age of CD diagnosis.

## Discussion

Our study indicated that CD is a common occurrence in the elderly, and the number of individuals being diagnosed over the age of 65 is rising. In our cohort, 12.9% of patients diagnosed during the 30-year period were over the age of 65 years at CD diagnosis. A similar proportion of elderly CD patients was identified in an American study which found that 12.4% of patients were diagnosed in old age, using a threshold of 65 years old (23) . Lohi et al. reported even a higher prevalence of elderly CD diagnosis in Finland. Nevertheless, the homogeneity of the Finnish population must be taken into account when considering these results (3) . 

The disparity in the proportion of elderly CD diagnoses may result from a low index of suspicion in some centres, resulting in delayed diagnosis. In this context, patients are often investigated for more serious conditions that can present in a similar fashion to CD. Anaemia is a particularly common mode of presentation in CD patients but is also highly indicative of colon cancer in the elderly, making such patients vulnerable to ‘missed’ diagnoses if duodenal biopsies are not collected (24) . This is particularly true in cases of milder mucosal damage in which the characteristic duodenal pathology of mucosal mosaicism as well as reduction and scalloping of duodenal folds is not macroscopically visible.

In 2016-2017, 41 CD diagnoses were made in individuals aged 65 and older, accounting for 18.7% of all CD cases identified during this period. This was a statistically significant increase from 1990-1991 when no elderly CD cases were reported. Casella et al. observed a similar trend in elderly coeliacs in Brescia, Italy, with the prevalence almost doubling between 2002 and 2012  (25) . On the other hand, an epidemiological study in Derby found an increase of 0.01% to 0.25% between 1984 and 2014 in those aged 60 years and over (26) . This was a prospectively recruited cohort exclusively incorporating patients born in the Derby catchment area, allowing accurate prevalence figures to be obtained. Vilppula et al found that the prevalence of CD in subjects over the age of 50 increased from 2.13% to 2.34% between 2002 and 2005 with a crude yearly incidence of 0.08% (27)  (28) . All these studies support a rise in the number of CD diagnoses made in advanced age individuals despite the large variations in the prevalence.

Whether or not these findings represent a true rise in elderly disease prevalence is contested. It is possible that this trend represents an increase in de-novo manifestation of coeliac disease in elderly adults who were previously gluten-tolerant. Lohi et al. proved that CD could manifest clinically for the first time in elderly individuals with previous evidence of complete tolerance to gluten ingestion (3) . It is well-established that the worldwide prevalence of autoimmune diseases as a whole has increased significantly over the past three decades, and the prevalence of autoimmune diseases increases with age, putting elderly individuals with a genetic predisposition to CD at a high risk (6) .

The diverse clinical spectrum of adult coeliac disease has resulted in heterogeneity in the classification of CD presentation in the medical literature. This study categorised the clinical presentation of CD as either malabsorption or non-malabsorption features regardless of “classical” or “non-classical” nomenclature.

The current study found a statistically significant association between age group at diagnosis and prevalence of B12 deficiency at presentation (p=0.017). The prevalence was significantly higher in the 65 and over age group (20.2%) than in the 16-34 and 35-64 age groups (10.5% and 10.8% respectively). This is in agreement with the findings of Freeman who identified a 37% prevalence of B12 deficiency in elderly coeliacs (29) . 

The large number of B12-deficient elderly CD patients in the present study is intriguing as vitamin B12 is primarily absorbed in the distal ileum via attachment to intrinsic factors. 

In contrast to vitamin B12, the predominant site of iron absorption is the proximal duodenum. The present study showed that 54% of patients over the age of 65 were anaemic at diagnosis. This supports previous studies suggesting a prevalence of 58-80% in elderly CD, with this haematological finding being the sole presenting feature in 22% of aged patients.

Iron-deficiency anaemia was more common amongst those diagnosed between 16 and 34 (34.5%) than those over 65 (29.3%), though this association was not statistically significant. This finding was unexpected due to the multitude of studies demonstrating the largely extraintestinal nature of elderly CD presentation. However, during later analyses, it was found that younger patients were significantly more likely to present with fatigue than elderly patients, and thus this result may reflect a higher rate of anaemia detection in younger patients due to greater symptomatic manifestation rather than a higher absolute prevalence. Crucially, CD-associated anaemia is a multifactorial condition occurring in patients of all ages, with underlying intestinal inflammation being only one contributing factor.

Casella et al. (30)  and Mukherjee et al. (23)  reported no association between the degree of duodenal inflammation and diagnostic age. The sporadic, patchy nature of villous damage may result in numerous missed diagnoses if biopsies are by chance collected from areas of undamaged bowel despite the presence of atrophic villi (31) . Further, elderly CD patients might have a more severe distal disease which is not accessible to conventional upper GI endoscopy; thus, capsule endoscopy may be warranted in these patients (4) . Indeed, histopathological interpretation of biopsy samples from the distal small bowel has suggested that intestinal damage may extend beyond the duodenum (32) .

Furthermore, elderly patients appeared to be less symptomatic than their younger counterparts, with micronutrient deficiencies, osteopenia, and osteoporosis being the predominant features prompting referral. In contrast, gastrointestinal manifestations including diarrhoea, abdominal pain, and IBS-type symptoms were significantly more prevalent in younger patients, though iron-deficiency anaemia was still the predominant presenting feature encouraging investigation. This may represent the largely asymptomatic nature of elderly CD, with patients being principally identified because of more regular serological screening. This is a likely possibility given that elderly patients are more commonly enrolled on ‘cancer pathways and metabolic bone screening’ programs.

In conclusion, considering the reduced presence of symptoms in the elderly population, the present study findings question the necessity of active case finding in elderly patients with CD. Is it worth attempting to break lifetime dietary habits in patients who are not symptomatic in the first place? It is possible that nutrient supplementation may be sufficient to manage patients presenting solely with micronutrient deficiencies.

Should such an approach is taken, a number of other factors need to be investigated further. The long-term risk of CD-associated complications such as small bowel lymphoma, anaemia, and osteoporosis need to be evaluated.

## Conflict of interests

The authors declare that they have no conflict of interest.
